# Mapping Strategies for Strengthening Safety Culture: A Scoping Review

**DOI:** 10.3390/healthcare12121194

**Published:** 2024-06-13

**Authors:** Cristiane de Lima Pacenko, Karla Crozeta Figueiredo, Elisabete Nunes, Paulo Cruchinho, Pedro Lucas

**Affiliations:** 1 Postgraduate Program in Nursing, Department of Nursing, Federal University of Paraná, Avenue Prefeito Lothário Meissner 632, Curitiba 80210-170, Brazil; karla.crozeta@ufpr.br; 2 Nursing Research, Innovation, and Development Centre of Lisbon (CIDNUR), Escola Superior de Enfermagem de Lisboa, Avenida Professor Egas Moniz, 1600-190 Lisboa, Portugal; enunes@esel.pt (E.N.); pjcruchinho@esel.pt (P.C.); prlucas@esel.pt (P.L.)

**Keywords:** patient safety, organizational culture, health strategies, safety management

## Abstract

Background: Twenty years after the “To Err Is Human” report, one in ten patients still suffer harm in hospitals in high-income countries, highlighting the need to strengthen the culture of safety in healthcare. This scoping review aims to map patient safety culture strengthening strategies described in the literature. Method: This scoping review follows the JBI methodology. It adhered to all scoping review checklist items (PRISMA-ScR) with searches in the Lilacs, MedLine, IBECS, and PubMed databases and on the official websites of Brazilian and North American patient safety organizations. The research took place during the year 2023. Results: In total, 58 studies comprising 52 articles and 6 documents from health organizations were included. Various strategies were identified and grouped into seven categories based on similarity, highlighting the need for a comprehensive organizational approach to improve patient care. The most described strategies were communication (69%), followed by teamwork (58.6%) and active leadership (56.9%). Conclusion: The identified strategies can promote the development of a culture in which an organization can achieve patient safety, involving practices and attitudes that reduce risks and errors in healthcare. However, the identification of strategies is limited because it is restricted to certain databases and websites of international organizations and does not cover a broader spectrum of sources. Furthermore, the effectiveness of these strategies in improving patient safety culture has not yet been evaluated.

## 1. Introduction

The updated definition of patient safety by the World Health Organization (WHO) highlights the importance of adopting preventative measures to consistently reduce risks and prevent harm, as well as minimize the consequences of that harm when it occurs. This definition highlights the need for a proactive and systematic approach to safety in healthcare [1]. The relevance of this issue is further highlighted when considering the Institute of Medicine’s landmark report from 2000, entitled “To Err Is Human: Building a Safer Health System”. This report was a landmark, raising awareness of the severity of errors and ushering in a new era of focus on patient safety as an essential component of quality healthcare [2,3]. Despite significant progress since the publication of this report, challenges persist. Some two decades later, it is still estimated that approximately one in ten patients in high-income countries suffer some type of harm while receiving hospital care. This statistic underscores the complexity of completely eradicating risks associated with healthcare and the importance of ongoing, effective strategies to improve patient safety, highlighting the need for a global, integrated approach to addressing these challenges. Therefore, patient safety remains a critical issue that requires continuous attention, research and implementation of best practices worldwide [2,3].

There is currently consensus in the literature on the need to support these initiatives to develop and improve patient safety culture to reduce the occurrence of incidents [4,5]. This culture encompasses attitudes, perceptions, values, individual and group competencies, and patterns of behavior that determine commitment, style, and proficiency regarding patient safety issues [4,5].

Therefore, health organizations worldwide advocate implementing practices and programs to strengthen patient safety culture. These strategies are used in experiences from high-reliability organizations, such as the nuclear and aviation industries, which involve high-risk operations but with few occurrences of events [6].

In this context, the objective of this scoping review is justified, which aims to map strategies for strengthening the patient safety culture described in the literature—a complex, multifaceted, and multidimensional topic that spans safety policies, healthcare professional training, and patient involvement, among other aspects. This review can provide valuable information for decision-making in healthcare services and patient safety policies, aiming to summarize the current state of knowledge and highlight promising strategies [7].

Furthermore, the novelty of this study lies in the compilation and analysis of contemporary and innovative strategies, which offers a unique contribution to the field of health security. This review adds value by consolidating practices and interventions that have not been widely discussed or integrated in previous reviews [7].

Strategies for strengthening patient safety culture refer to planned and targeted approaches to promote an organizational culture where safety is valued, incorporated, and practiced by all members of an organization. These strategies aim to create an environment in which safety is a priority and is integrated into daily processes, behaviors, and decisions [8,9,10,11].

## 2. Materials and Methods

This scoping review followed the JBI methodology following the PRISMA-ScR Checklist (Preferred Reporting Items for Systematic reviews and Meta-Analyses extension for Scoping Reviews): problem and research question formulation; data collection; data analysis and interpretation; data categorization; and presentation of results and conclusions [7].

### 2.1. Protocol and Registration

The protocol was developed and registered on the Open Science Framework (OSF) repository in June 2023, available at: https://osf.io/edtc6/, https://doi.org/10.17605/OSF.IO/EDTC6 (accessed on 10 January 2024).

### 2.2. Inclusion and Exclusion Criteria

The PCC strategy—an acronym for population (all health professionals, such as doctors, nurses, nursing assistants, among others), concept (strategies for strengthening the patient safety culture), and context (health services, such as primary healthcare, hospitals, and long-term care, among others)—was used to define the research question. The guiding question for this review was “What are the strategies for strengthening the patient safety culture in healthcare services described in the literature?” The review aimed to map the strategies for strengthening patient safety culture described in the literature.

A prior review was carried out on the OSF platform with the aim of identifying completed or ongoing review protocols on this topic, but no results were obtained.

Regarding the studies, those that addressed the guiding question were included, if they were without language restrictions, published in the last ten years, in full, freely available in journals accessible through the selected databases, and consistent with the proposed objective and with the descriptors listed in the search. Restricted-access databases can be accessed through computers at the Federal University of Paraná or via home connection through the CAFe Network and VPN/UFPR Remote Connection. Studies related to patient safety culture that did not describe strategies and/or tools for strengthening patient safety culture were excluded.

The inclusion criteria for documents from Brazilian and North American health organizations (patient safety organizations in these regions are leaders in innovation and safety policy development, which can provide valuable insights into best practices and emerging trends) were as follows: documents available on the researched websites, with content specifically focused on the proposed theme, in leading organizations or that influenced the thematic areas of the review, contributed to innovative research, policies, or practices and were relevant. The exclusion criteria comprised documents that did not describe strategies and/or tools for strengthening patient safety culture, including those related to patient safety but not those related to patient safety culture.

### 2.3. Search Strategy

The research was carried out in three stages. In the first stage, a search was carried out in the PubMed database to identify descriptors and keywords related to the topic. In the second stage, these were applied to the databases consulted, with due adaptation. The third phase consisted of analyzing the bibliographic references of the selected documents to retrieve potentially relevant documents.

In national and international publication databases, the search took place in November 2022, with study selection in the following databases: the Latin American and Caribbean Health Sciences Literature (Lilacs), the Medical Literature Analysis and Retrieval System Online (MedLine), the Spanish Bibliographic Index in Health Sciences (IBECS), and the National Library of Medicine National Institutes of Health (PubMed). LILACS and IBECS specialize in the scientific literature from Latin America, the Caribbean, and Spain. This choice ensured that the research included a regional perspective that may be less represented in other broader international databases, providing a more complete view of patient safety practices in these specific regions. The selection of these databases allows access to studies published in Spanish and Portuguese, the predominant languages in Latin America and Spain. This is crucial for capturing cultural and linguistic nuances that can influence patient safety practices and public health policies in these areas. The temporal cutoff was the last ten years to track the evolution of strategies used and identify the most recent and effective ones because patient safety is a field in constant evolution, with new research, discoveries, and practices emerging over time. Thus, it is convenient to identify and analyze the most current strategies that have been successfully implemented to strengthen patient safety culture. These strategies likely reflect current best practices and provide valuable insights for improving patient safety in the present and future.

The search strategy was developed with the assistance of a librarian (Table 1). MeSH (Medical Subject Headings) descriptors were used in four languages, Portuguese, English, Spanish, and French, along with Boolean operators represented by “AND” (restrictive combination) and/or “OR” (additive combination).

The search was conducted in November 2022 and updated in December 2023 on the Virtual Health Library (BVS) platform, applying filters from the following databases: Lilacs, MedLine, IBECS, and a search on PubMed.

In the search in PubMed, we did not insert any keywords relating to health services or health professionals because the search expression mainly used MesH descriptors, which are, by definition, specific to the health area, for example, the descriptor “organizational culture”, which is within the category “Organization and Administration” and is within the broader category “Health Services Administration”. The descriptor “safety management” belongs to the “risk management” category, which is also within the “Health Services Administration” category.

Searches on the official websites of patient safety organizations were conducted for organizations pioneering the topic of patient safety, including the World Alliance for Patient Safety (WAPS/WHO), the Agency for Healthcare Research and Quality (AHRQ), the Institute for Healthcare Improvement (IHI), the National Steering Committee for Patient Safety (NSC), Joint Commission International (JCI), and the National Patient Safety Agency (NPSA), in addition to Brazilian organizations such as the Brazilian Institute for Patient Safety (IBSP), the Brazilian Society for Quality of Care and Patient Safety (SOBRASP), and the Patient Safety Foundation (FSP).

In healthcare organizations, the research period was between October and November 2022, with an update in December 2023, using the term “strategies for strengthening the patient safety culture” in the search field of the websites. Furthermore, document searches were initially conducted in Portuguese and later in English.

### 2.4. Study Selection

All articles were imported into Rayyan (a tool specifically developed to support the selection and inclusion of studies in reviews) for study selection based on titles and abstracts [12]. Two independent reviewers read and analyzed the data to identify those potentially eligible for the study, and a third independent reviewer resolved any conflicts of opinion.

To confirm the relevance of the research question, two reviewers read the selected studies in full.

### 2.5. Data Extraction

For data extraction, two Excel spreadsheets were created following the JBI data extraction model. The first comprised the following synthesis columns: title, author, year, language, and journal. The second synthesized the selected strategies, arranged in rows, while the columns represented the articles and organizations that were sources of these strategies.

To identify the strategies, the concept of an implementation strategy from the Taxonomy of Interventions in Health Systems of Effective Practice and Organization of Care (EPOC) was used, which defined implementation strategies as interventions designed to bring about changes in health organizations, the behavior of health professionals, or the use of health services by healthcare recipients [13].

This approach allowed for a clear and systematic organization of information, facilitating the analysis and understanding of the proposed strategies in each source. By structuring the strategies in a spreadsheet, it became easier to identify trends, similarities, and differences in the recommended strategies and proposed actions. Additionally, it provided an overview of the different perspectives and approaches adopted by various authors and organizations, contributing to a more comprehensive and informed analysis.

### 2.6. Evidence Analysis

For the analysis of the results and the final interpretation of the obtained data, the content analysis (CA) method proposed by Bardin was utilized [14].

Through preanalysis, superficial reading of all included articles and documents from databases and organizations was conducted to organize the selected material. Subsequently, there was an exploration of the material and data treatment, involving an exhaustive reading of the materials, breaking down the texts into categories due to the diversity of strategies found. This included identifying keywords for categorizing the strategies.

The selected strategies were listed and grouped based on similarities, such as training for leaders and team training grouped under the strategy of education/training system. The list of strategies was organized into two categories: (1) recommendations and (2) actions.

Recommendations in patient safety culture refer to guidelines based on evidence or expert consensus intended to guide practices, policies, or behaviors to promote patient safety within healthcare organizations. Actions refer to specific measures, strategies, or interventions implemented to meet the recommendations. These actions are practical and targeted and are designed to effect concrete changes to patient safety culture. They include implementing training programs for healthcare professionals on how to report and learn from errors, developing incident reporting systems that support open and nonpunitive communication, and promoting initiatives that strengthen management support for safe practices [15].

To construct groups of strategies based on their proximity, principal component analysis (PCA) was applied using R 4.2.2 software. This methodology allows the measurement of phenomena that are not directly observable, such as the importance of strategies according to the frequency with which they were used in the articles. PCA is used to identify latent constructs and aims to reduce the original information to a smaller set of factors, known as “loadings”. These loadings represent the latent dimensions (constructs) that summarize the original set of variables while maintaining the representativeness of the characteristics of the original variables [16].

In this study, PCA resulted in sets of loadings. This method constructs a cluster tree, also known as a “dendrogram”, in which objects are organized in hierarchical levels, reflecting their similarity to each other. Finally, grouping was performed based on the level of similarity among the located strategies, identifying groups of strategies that are more similar to each other at different levels of granularity.

## 3. Results

The search resulted in 139 articles in BVS (with 117 in MEDLINE, 17 in LILACS, and 5 in IBCS) and 75 articles in PubMed, totaling 214 articles. Of these, 8 duplicate articles were excluded. In the preselection phase, 129 articles were excluded based on title reading, and 20 had conflicting opinions. A third reviewer was invited for resolution, and 18 articles were excluded, leaving 59 articles for full-text reading.

After reading the complete texts, five articles that did not present any strategies for strengthening patient safety culture were excluded. One duplicated article that needed to be manually excluded after reading, as the duplicate was not identified in the preselection due to publication in two languages (English and Portuguese), and one article that did not address patient safety culture was excluded, for a total of 52 articles.

On the websites of healthcare organizations, seven documents addressing strategies for strengthening patient safety culture were found. The document from the BC Patient Safety & Quality Council (BCPSQC) was excluded as a duplicate, as it was translated into Portuguese on the SOBRASP website, totaling six documents.

Thus, 58 documents were included in the review for analysis and data extraction. No further documents were recovered through the analysis of bibliographic references.

**Figure 1:**
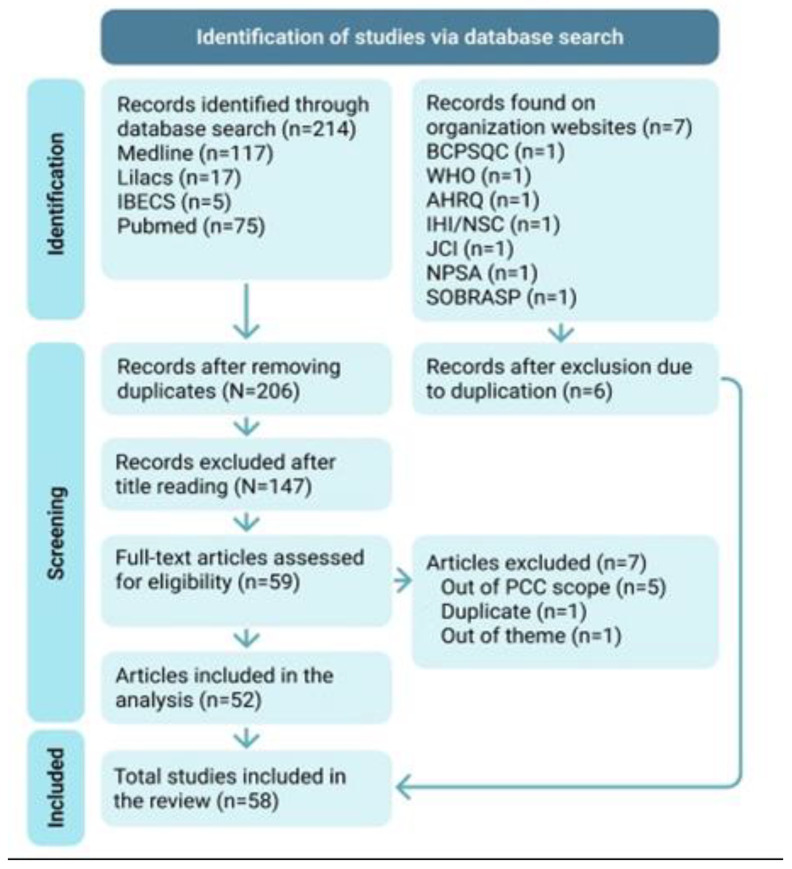
Modified PRISMA 2020 flow diagram for studies included within this scoping review. Subtitle: AHRQ—Agency for Healthcare Research and Quality; IBECS—Spanish Bibliographic Index in Health Sciences; IHI—Institute for Healthcare Improvement; JCI—Joint Commission International; Lilacs—Latin American and Caribbean Health Sciences Literature; MedLine—Medical Literature Analysis and Retrieval System Online; NSC—National Steering Committee for Patient Safety; NPSA—National Patient Safety Agency; PubMed—National Library of Medicine National Institutes of Health; SOBRASP—Brazilian Society for Quality of Care and Patient Safety; WHO—World Health Organization.

Figure 1 presents the flowchart of the publication selection process included in this review.

### Characteristics of the Studies

The 52 selected scientific articles were identified and labeled with the numbers 6 and 17 to 67 (Table 2), while the 6 documents from national and international patient safety organizations are listed in Table 3.

The studies were mostly published between 2015 and 2019, with the highest number occurring in 2017. English was the predominant language (48), followed by Portuguese (2), Spanish (1), and German (1). The main journals that published them were the *Joint Commission Journal on Quality and Patient Safety* (2), *J. Healthc Risk Manag.* (2), *BMJ Open* (2), *The Journal of Nursing Administration* (2), *BMJ Quality & Safety* (2), and *J. Healthc Risk Manag.* (2).

The results revealed 57 strategies, which were identified and categorized into 21 recommendations and 36 actions that are presented in Table 4, with their respective concepts and frequencies appearing in the documents.

The most frequently mentioned strategies were related to communication (40), followed by teamwork (34) and active leadership (33). Regarding the frequency of strategies cited in each article, on average, each article mentioned approximately seven different strategies.

In the recommendations category, 21 strategies were listed in the 58 analyzed studies, with communication being the most prevalent at 69%, followed by teamwork at 58.6%, and active leadership at 56.9%.

In the actions category, 36 strategies were described, with 21 types of strategies having only one citation, corresponding to 1.72% each, meaning that 58.3% appeared in only one study. The most prevalent action strategy was team engagement at 20.6%, followed by meetings/group dynamics at 15.5% and realistic simulation at 13.7%.

**Figure 2:**
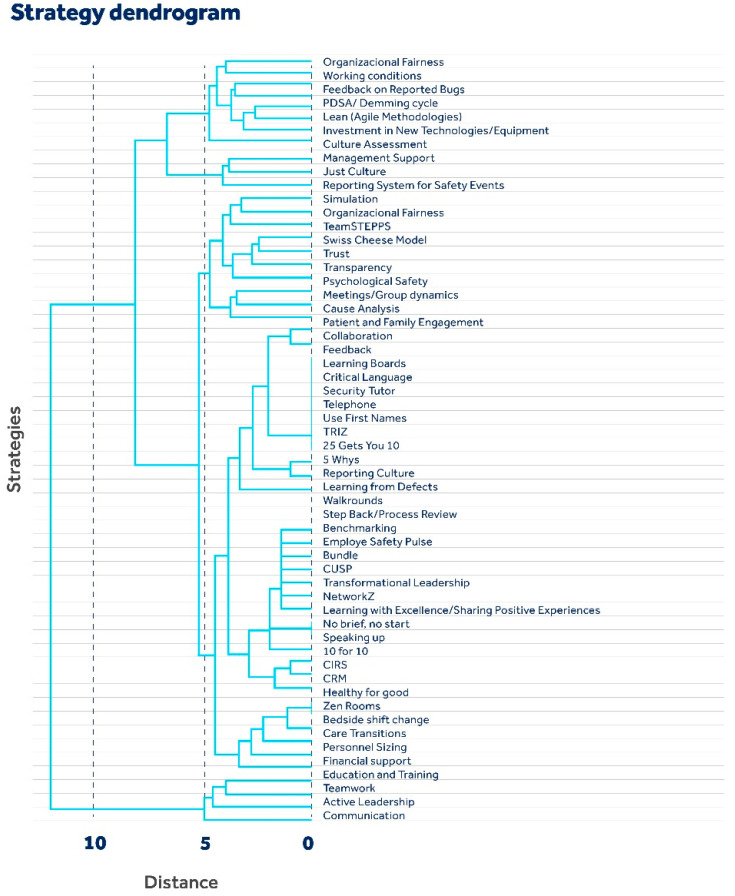
Hierarchical clustering dendrogram of strategies.

In the analysis to group the strategies based on their similarities, i.e., to determine how many articles they appear together in, the hierarchical dendrogram serves as a fundamental visual and analytical tool, as shown below in Figure 2.

The dendrogram is a visual representation of the similarities between different strategies for strengthening safety culture. It is built using a hierarchical clustering algorithm, which groups strategies based on their similarities, forming a tree-like structure.

The horizontal axis (distance) represents the distance or dissimilarity between strategies. The greater the distance on the horizontal axis is, the less similar the strategies are. Vertical lines connect strategies at different levels of similarity. The vertical axis (strategies) lists the specific strategies that have been grouped. Each leaf of the dendrogram represents an individual strategy.

According to the dendrogram structure, strategies appearing together in many articles are linked by lower branches, indicating a strong relationship or a common pattern of strategy adoption within the studied field.

There are distinct levels of grouping visible, where some strategies are more closely related to each other than others. For example, “Trust”, “Transparency”, and “Psychological Safety” are grouped together at a low level of the dendrogram, which suggests that these strategies share greater similarity or are more often implemented together. Strategies that come together at the top of the dendrogram, such as “Bedside shift change” and “Care transitions”, indicate that although they share some similarity, they have significant differences compared to groups that come together more closely below.

Organizational protocols, feedback on reported errors, and patient and family engagement appear to be key strategies. These are fundamental to creating a culture in which patient safety is prioritized. The proximity of these strategies in the dendrogram suggests a strong interconnection between them.

The chart can also help determine which strategies could be adopted together or which could require different approaches. For example, when looking to improve communication within an organization, strategies that are close together on the dendrogram, such as “Telephone” and “Use First Names”, may be more effective when implemented together.

When analyzing the articles with the aim of grouping the strategies based on their similarities, that is, to determine how many articles they appear together, it is noted that articles 37 and 40 are directly connected. It is inferred that the strategies (teamwork, communication, active leadership, psychological safety, and educational systems) discussed in these articles have a high incidence of co-occurrence, suggesting that, in the literature, these strategies are often considered together or perhaps complement each other.

The strategies discussed in organization 1 (transparency, communication, active leadership, fair working conditions, and just culture) and in article 48 (teamwork, active leadership, shift transfer and transition issues, patient and family engagement, culture assessment, financial support, meetings/group dynamics, PDSA cycle, and bedside passage) occur at greater distances than most other pairs, which allows us to infer that there is a distinct relationship between patient safety strategies discussed in this article and those adopted or recommended by organization document 1.

Additionally, the presence of articles or documents from organizations such as organization 69 may indicate unique or new approaches to patient safety culture that are not widely discussed or have not yet been integrated into mainstream literature. This may point to emerging areas of research or innovative strategies that deserve additional attention.

Articles 46 and 57 show a strong positive correlation, suggesting that the strategies discussed are frequently mentioned or used together, both bringing to light communication strategies, fair culture, and notification systems. Articles 47 and 64 also mention common strategies.

The analysis of articles 18 and 33 suggested that the strategies discussed in these two articles are not strongly related or may even be inversely related.

A negative correlation may suggest that practices or strategies from one article are rarely implemented in conjunction with those from another or that the organizations’ approaches differ significantly.

Finally, in the grouping by the level of similarity between located strategies, seven groups were described, represented below:

Group 1: Organizational Principles and Culture: This group includes key principles such as organizational justice, transparency, trust, a Zen room, and just culture. These elements are fundamental to creating a healthy and ethical organizational culture. They establish the foundation for an environment where healthcare professionals can feel valued, respected, and confident in their ability to perform their duties. A just and transparent culture promotes accountability and reliability, which is crucial for patient safety.

Group 2: Leadership and Personal Development: Strong leadership is a central component of safety culture. This group addresses the development of leadership skills and the promotion of a learning culture in which professionals are encouraged to learn from their mistakes and report incidents openly and responsibly. This group includes the strategies security tutor, reporting culture, teamwork, learning from defects, active leadership, psychological safety, learning boards, working conditions, feedback, management support, NetworkZ, transformational leadership, and patient and family engagement.

Group 3: Education and Continuous Improvement: This group encompasses elements such as education/training systems, assessment of culture/strategies, cause analysis, feedback on reported bugs, organizational protocols, meetings/group dynamics, and speaking up. The continuous education of healthcare professionals is crucial for keeping them up-to-date and skilled. Culture assessment and root cause analysis help to identify problematic areas and opportunities for improvement. Feedback on errors allows learning from failures and implementing changes to avoid repeating mistakes in the future.

Group 4: Technology and Resources: Topics related to investments in technology and financial resources are included. The incorporation of new technologies and equipment can improve process efficiency and treatment accuracy. Adequate financial support is essential to ensure that the necessary resources are available to provide the best possible care to patients. The strategies included in this group are investment in new technologies/equipment, financial support, and reporting systems for safety events.

Group 5: Process improvement and safety: This group addresses specific tools and strategies to improve patient safety. Standardizing processes, identifying care transition issues, and implementing safety protocols are vital to minimizing errors and adverse events. Lean practices and other continuous improvement methodologies also help optimize processes and reduce waste. The following strategies are included: CRM, CIRS, simulation, staff sizing, care transitions, bedside handover, bundle, CUSP, step back and lean.

Group 6: Communication and Decision-Making: Effective communication is essential in all aspects of patient care. This group covers tools and techniques to improve communication, such as the use of first names and assertive communication techniques. These approaches help ensure that critical information is conveyed clearly and that decisions are made based on accurate data. This group includes communication, critical language, telephoning, using first names, TRIZ, 25 gets you 10, 5 whys, and collaboration.

Group 7: Improvement Strategies and Interventions: This group describes strategies and interventions that can be used to drive continuous improvement. This includes benchmarking, PDSA, and team involvement in the improvement process. There are also 10 respondents in this group: Walkrounds, TeamSTEPPS, the Swiss cheese model, Employee Safety Pulse, learning with excellence, and the health for good program. Such strategies provide a framework for identifying areas of opportunity and implementing effective changes.

## 4. Discussion

Patient safety culture refers to the organizational environment in a healthcare institution, where emphasis is placed on patient safety and well-being. An effective safety culture promotes a proactive approach to identifying, reporting, and preventing errors, adverse incidents, and adverse events, aiming to provide high-quality healthcare and minimize risks [73].

Therefore, culture is how we collaborate; collaboration is not just an activity but an expression of shared values, norms, and behaviors that define and are defined by the cultural environment in question [1]. Although it may seem challenging, the good news is that each of us, both individually and collectively, can promote it [68].

When we map strategies to strengthen patient safety culture, the objective of this review, we take an important step in improving healthcare safety. This involves the identification and categorization of practices and approaches that can be implemented to promote a strong safety culture. This is demonstrated in the study carried out in 2020 by Armutlu et al., in which a set of evidence-based practices was constructed that must be applied collectively to establish and maintain a culture of quality and safety in order to provide safe care [58].

In this research, scientific database searches and web-based activities tracked information relevant to the search results. Healthcare organizations and their publications on patient safety have been an enriching research environment, as they aim to contribute to improving healthcare decision-making based on the best available information, as we can see in the documents Culture Change Toolbox [68], Action Planning Tool for the AHRQ Surveys on Patient Safety Culture [69], Safer Together: A National Action Plan to Advance Patient Safety [70], Sentinel Event Alert 57: The essential role of leadership in developing a safety culture [71], The Incident Decision Tree: Guidelines for Action Following Patient Safety Incidents [72] and the Global Patient Safety Action Plan 2021–2030 [1].

Both research methods involved strategies targeting a diverse range of issues, including organizational principles, leadership, teamwork, continuous education, communication, and technologies. These areas are crucial for promoting a safe and collaborative environment in healthcare contexts. For example, effective communication is fundamental for avoiding errors, while teamwork and active leadership are essential for the efficient implementation of safety procedures and fostering a culture of shared responsibility and continuous learning.

Categorizing strategies into recommendations and actions is important for a future practical implementation approach. Recommendation strategies may include general principles or guidelines to enhance safety culture, while action strategies are specific practices that can be applied to achieve these objectives.

The correlation analyses carried out help to identify which strategies are commonly adopted together, which can be valuable for developing more integrated and comprehensive policies and training programs. For example, if strong leadership and effective communication are frequently correlated, focusing on both simultaneously may be an effective practice.

The fact that 21 strategies were mentioned only once, representing 58.3% of the total strategies appearing in only one study, suggests a wide range of specific or punctual approaches within the field, highlighting the complex and multifaceted nature of patient safety culture and suggesting that these can be explored in future research.

The representativeness of the group with the seven strategic groups provided a comprehensive approach to improving patient care. Implementing these strategies may contribute to prioritizing patient safety across all areas of healthcare. This will help create an environment where healthcare professionals feel empowered to report issues, learn from past incidents, and take measures to prevent errors.

The strength of this review lies in the innovative analysis of the literature, citing important recommendations for strategies to improve patient safety culture. The identified strategies can promote the development of a culture in which an organization can achieve patient safety, involving practices and attitudes that reduce risks and errors in healthcare. This makes the study relevant and useful for health professionals and managers.

### Limitations

This research was limited to identifying strategies and did not assess the effectiveness of these strategies in improving patient safety culture. Many studies in the literature present these strategies as recommendations, but their effectiveness in practice may vary.

Research is recommended to verify the effectiveness of the strategies listed in the literature in clinical practice. This suggests that research should not be limited to identifying strategies but should be accompanied by practical efforts to incorporate them into healthcare routines.

## 5. Final Considerations

The scoping review identified 57 strategies that can be used to support management in strengthening patient safety culture, involving practices and attitudes that reduce risks and errors in healthcare. This highlights the diversity of approaches available in the field of patient safety, indicating that there is no single or universally applicable approach, and healthcare organizations can choose from a wide range of strategies to meet their specific needs and the context in which they operate.

The strategies were divided into two main categories: recommendations and actions. In the recommendations group, 21 strategies were mentioned, with significant emphasis on those related to communication, teamwork, and active leadership. In the actions group, 36 strategies were listed, with a focus on team engagement, which reflects the importance of involving healthcare professionals in promoting patient safety, thereby encouraging active participation and commitment to safety practices.

The research results provide a valuable overview of current practices and have the potential to guide future research and practical implementations in the field of patient safety. This, in turn, can contribute to creating a safer and more efficient healthcare environment, benefiting both patients and healthcare professionals.

## Figures and Tables

**Table 1:** Search strategies with the use of descriptors, entry terms, and Boolean operators, according to the databases.

Latin American and Caribbean Health Sciences Literature (Lilacs) Medical Literature Analysis and Retrieval System Online (MedLine) Spanish Bibliographic Index in Health Sciences (IBECS)
(Pessoal de Saúde) or (Personal de Salud) or (Health Personnel) or (Segurança do Paciente) OR (Patient Safety) OR (Patient Safeties) OR (Seguridad del Paciente) OR (Sécurité des patients) OR (Sécurité des patientes) OR (Sécurité du patient) AND (Gestão da Segurança) OR (Gerenciamento de Segurança) OR (Administração da Segurança) OR (Administração de Segurança) OR (Safety Management) OR (Administración de la Seguridad) OR (Administración de Seguridad) OR (Gestión de la Seguridad) OR (Gestión de Seguridad) OR (Gestion de la sécurité) OR (Culture de la sécurité) AND (Estratégias de Saúde) OR (Health Strategies) OR (Estrategias de Salud) OR (Stratégies de Santé) AND (Cultura Organizacional) OR (Organizational Culture) OR (Cultura Organizacional) OR (Culture organisationnelle) AND (Pacotes de Assistência ao Paciente) OR (Patient Care Bundles) OR (Paquetes de Atención al Paciente) OR (Bouquets de soins despatients) OR (Health Services) OR (Serviços de Saúde) OR (Servicios de Salud)
**National Library of Medicine National Institutes of Health (PubMed)**
“Management” AND “Safety Management/methods” OR “Safety Management/organization and administration” OR “Safety Management/trends” AND “Organizational Culture”

**Table 2:** Identification of all selected articles in the review.

Title [Reference]	Author	Journal (Year)	Language	Kind of Study
Researching safety culture: Deliberative dialog with a restorative lens [6].	Lorenzini E, Oelke ND, MarckPB, Dall’agnol CM.	*Int J Qual Health Care* (2017)	English	Methodological study with deliberative dialog methods
What interventionalists can learn from the aviation industry [17].	Byrne RA	*Euro Intervention* (2018)	English	Interview
Human factors and crisis resource management: improving patient safety. [18].	Rall M, Oberfrank S	*Unfallchirurg* (2013)	German	Opinion article
What regulations have launched autonomous communities to going forward on patient safety culture in healthcare organizations? [19].	Romeo Casabona CM, Urruela Mora A, Peiró Callizo E, Alava Cano F, Gens Barbera M, Iriarte Aristu I et al.	*J Healthc Qual Res* (2019)	Spanish	Descriptive study
A multilevel neo-institutional analysis of infection prevention and control in English hospitals: coerced safety culture change? [20].	Kyratsis Y, Ahmad R, Iwami M, Castro-Sánchez E, Atun R, Holmes AH.	*Sociology of health & disease* (2019)	English	Case study
Apparent Cause Analysis: A Safety Tool [21].	Parikh K, Hochberg E, Cheng JJ, Lavette LB, Merkeley K, Fahey L et al.	*Pediatrics* (2020)	English	Case study
Applying an ecological restoration approach to study patient safety culture in an intensive care unit [22].	Gimenes FRE, Torrieri MCGR, Gabriel CS, Rocha FLR, Silva AEB de C, Shasanmi RO et al.	*J Clin Nursing* (2016)	English	Exploratory research
Association Between Implementing Comprehensive Learning Collaborative Strategies in a Statewide Collaborative and Changes in Hospital Safety Culture [23].	Ford EW, Silvera GA, Kazley AS, Diana ML, Huerta TR.	*JAMA Surg* (2016)	English	Multilevel study
Building a culture of safety through team training and engagement [24].	Thomas L, Galla C.	*BMJ Qual Saf* (2013)	English	Organizational implementation case study
Building Safe, Highly Reliable Organizations: CQO Shares Words of Wisdom [25].	Wyatt R.	*Biom Instrum Technol* (2017)	English	Expert opinion study
Changing Operating Room Culture: Implementation of a Postoperative Debrief and Improved Safety Culture [26].	Magill ST, Wang DD, Rutledge WC, Lau D, Berger MS, Sankaran S et al.	*World Neurosurg* (2017)	English	Intervention study
Combining Systems and Teamwork Approaches to Enhance the Effectiveness of Safety Improvement Interventions in Surgery: The Safer Delivery of Surgical Services (S3) Program [27].	McCulloch P, Morgan L, New S, Catchpole K, Roberston E, Hadi M et al.	*Ann Surg* (2017)	English	Comparative Intervention Study
Creating a Culture of Safety Within an Institution: Walking the Walk [28].	Chera BS, Mazur L, Adams RD, Kim HJ, Milowsky MI, Marks LB.	*J Oncol Pract*. (2016)	English	Opinion article
Patient safety culture from the perspective of the multiprofessional team: an integrative review [29].	Alves DFB, Lorenzini E, Cavalheiro KA, Schmidt CR, Dal Pai S, Kolankiewicz ACB.	*R Pesq Cuid Fundam online* (2021)	English	Integrative review
Cultural Transformation After Implementation of Crew Resource Management: Is It Really Possible? [30].	Hefner JL, Hilligoss B, Knupp A, Bournique J, Sullivan J, Adkins E, Moffatt-Bruce SD.	*Am J Med Qual*. (2017)	English	Intervention study
Developing a culture of safety in an imaging department [31].	Pressman BD, Roy LT	*J Am Coll Radiol*. (2015)	English	Descriptive study
Developing person-centred analysis of harm in a paediatric hospital: a quality improvement report [32].	Lachman P, Linkson L, Evans T, Clausen H, Hothi D.	*BMJ Qual Saf*. (2015)	English	Experience report
Employee Engagement and a Culture of Safety in the Intensive Care Unit [33].	Collier SL, Fitzpatrick JJ, Siedlecki SL, Dolansky MA.	*J Nurs Adm*. (2016)	English	Retrospective
Enhancing Safety Culture Through Improved Incident Reporting: A Case Study In Translational Research [34].	Flott K, Nelson D, Moorcroft T, Mayer EK, Gage W, Redhead J, Darzi AW.	*Health Aff (Millwood)* (2018)	English	Intervention Study
Effective communication strategies for managing disruptive behaviors and promoting patient safety [35].	Moreira FTLS, Callou RCM, Albuquerque GA, Oliveira RM.	*Rev Gaúcha Enferm.* (2019)	Portuguese	Case study
Evolution of Culture on Patient Safety in the Clinical Setting of a Spanish Mutual Insurance Company: Observational Study between 2009 and 2017 Based on AHRQ Survey [36].	Ulibarrena MA, Vicunã LS, García-Alonso I, Lledo P, Gutiérrez M, Ulibarrena-García A et al.	*Int J Environ Res Public Health* (2021)	English	Transversal
Factors Influencing the Implementation of a Hospitalwide Intervention to Promote Professionalism and Build a Safety Culture: A Qualitative Study [37].	McKenzie L, Shaw L, Jordan JE, Alexander M, O’Brien M, Singer SJ et al.	*Jt Comm J Qual Patient Saf* (2019)	English	Case study
Frequency of and predictors for withholding patient safety concerns among oncology staff: a survey study [38].	Schwappach DLB, Gehring K	*Eur J Cancer Care* (Engl). (2015)	English	Cross-sectional quantitative research
Gating the holes in the Swiss cheese (part I): Expanding professor Reason’s model for patient safety [39]	Seshia SS, Bryan Young G, Makhinson M, Smith PA, Stobart K, Croskerry P.	*J Eval Clin Pract.* (2018)	English	Theoretical essay
Health care huddles: managing complexity to achieve high reliability [40].	Provost SM, Lanham HJ, Leykum LK, McDaniel RR Jr, Pugh J.	*Health Care Manage Rev.* (2015)	English	Qualitative exploratory
High reliability in healthcare: creating the culture and mindset for patient safety [41].	Cochrane BS, Hagins M Jr, Picciano G, King JA, Marshall DA, Nelson B, Deao C.	*Healthc Manage Forum.* (2017)	English	Descriptive/Exploratory
Impact of teamwork improvement training on communication and teamwork climate in ambulatory reproductive health care [42].	Dodge LE, Nippita S, Hacker MR, Intondi EM, Ozcelik G, Paul ME.	*J Healthc Risk Manag.* (2019)	English	Prospective Intervention Study
Improving Patient Safety Culture in Primary Care: A Systematic Review [43].	Verbakel NJ, Langelaan M, Verheij TJ, Wagner C, Zwart DL.	*J Patient Saf* (2016)	English	Literature review
Latent risk assessment tool for health care leaders [44].	Paine LA, Holzmueller CG, Elliott R, Kasda E, Pronovost PJ, Weaver SJ et al.	*J Healthc Risk Manage* (2018)	English	Analytical/Exploratory
Leading change to create a healthy and satisfying work environment [45].	Sanders CL, Krugman M, Schloffman DH.	*Nurs Adm Q* (2013)	English	Descriptive/evaluative
Leveraging a Safety Event Management System to Improve Organizational Learning and Safety Culture [46].	Dawson R, Saulnier T, Campbell A, Godambe SA.	*Hosp Pediat* (2022)	English	Prospective Intervention Study
Making an “Attitude Adjustment”: Using a Simulation-Enhanced Interprofessional Education Strategy to Improve Attitudes Toward Teamwork and Communication [47].	Wong AHW, Gang M, Szyld D, Mahoney H.	*Simul Healthc* (2016)	English	Observational Intervention Study
National Quality Program Achieves Improvements in Safety Culture and Reduction in Preventable Harms in Community Hospitals [48].	Frush K, Chamness C, Olson B, Hyde S, Nordlund C, Philips H et al.	*Jt Comm J Qual Patient Saf* (2018)	English	Retrospective case report
Near-misses are an opportunity to improve patient safety: adapting strategies of high reliability organizations to healthcare [49]	Van Spall H, Kassam A, Tollefson TT.	*Curr Opin Otolaryngol Head Neck Surg.* (2015)	English	Systemic review
Patient feedback for safety improvement in primary care: results from a feasibility study [50].	Hernan AL, Giles SJ, Beks H, McNamara K, Kloot K, Binder MJ et al.	*BMJ Open* (2020)	English	Mixed Methods Feasibility Trial
Patient Safety Culture Bundle for CEOs and Senior Leaders [51].	Armutlu M, Davis D, Doucet A, Down A, Schierbeck D, Stevens P.	*Healthc Q.* (2020)	English	Literature review
Patient safety culture: finding meaning in patient experiences [52].	Bishop AC, Cregan BR.	*Int J Health Care Qual Assur* (2015)	English	Qualitative exploratory research
Perception of the multiprofessional team regarding the safety of pediatric patients in critical areas [53].	Pereira FS, Silveira MS, Hoffmann LM, Peres MA, Breigeiron MK, Wegner W.	*Rev Enferm UFSM.* (2021)	Portuguese	Qualitative exploratory descriptive
Personal, situational and organizational aspects that influence the impact of patient safety incidents: A qualitative study [54].	Van Gerven E, Deweer D, Scott SD, Panella M, Euwema M, Sermeus W et al.	*Rev Calid Asist* (2016)	English	Qualitative exploratory
Priorities Related to Improving Healthcare Safety Through Simulation [55].	Paige JT, Terry Fairbanks RJ, Gaba DM.	*Simul Healthc*. (2018)	English	Conceptual and exploratory review
Remembering to learn: the overlooked role of remembrance in safety improvement [56].	Macrae C.	*BMJ Qual Saf*. (2017)	English	Narrative review
Safety culture includes “good catches” [57].	Traynor K	*Am J Health Syst Pharm*. (2015)	English	Expert opinion
Systematic implementation of clinical risk management in a large university hospital: the impact of risk managers [58].	Sendlhofer G, Brunner G, Tax C, Falzberger G, Smolle J, Leitgeb K et al.	*Wien Klin Wochenschr* (2015)	English	Evaluative case study
Targeting the Fear of Safety Reporting on a Unit Level [59].	Copeland D.	*J Nurs Adm*. (2019)	English	Intervention Study
Teams, tribes and patient safety: overcoming barriers to effective teamwork in healthcare [60].	Weller J, Boyd M, Cumin D.	*Postgrad Med J* (2014)	English	Literature review
The Impact of a 22-Month Multistep Implementation Program on Speaking-Up Behavior in an Academic Anesthesia Department [61].	Walther F, Schick C, Schwappach D, Kornilov E, Orbach-Zinger S, Katz D et al.	*J Patient Saf*. (2022)	English	Intervention Study
Tools for primary care patient safety: a narrative review [62].	Spencer R, Campbell SM.	*BMC Fam Pract*. (2014)	English	Narrative review
Towards a safer culture: implementing multidisciplinary simulation-based team training in New Zealand operating theatres—a framework analysis [63].	Jowsey T, Beaver P, Long J, Civil I, Garden AL, Henderson K et al.	*BMJ Open*. (2019)	English	Experience report
Transformational leadership in nursing: a concept analysis [64].	Fischer SA.	*J Adv Nurs* (2016)	English	Concept analysis
Understanding Facilitators and Barriers to Care Transitions: Insights from Project ACHIEVE Site Visits [65].	Scott AM, Li J, Oyewole-Eletu S, Nguyen HQ, Gass B, Hirschman KB, Mitchell S et al.	*Jt Comm J Qual Patient Saf* (2017)	English	Qualitative exploratory research
Walkrounds in practice: corrupting or enhancing a quality improvement intervention? A qualitative study [66].	Martin G, Ozieranski P, Willars J, Charles K, Minion J, McKee L et al.	*Jt Comm J Qual Patient Saf* (2014)	English	Empirical study
Weaving a culture of safety into the fabric of nursing [67].	Echevarria IM, Thoman M.	*Nurs Manage* (2017)	English	Experience report

**Table 3:** Documents from organizations selected for the review.

Document [Reference]	Agency and Acronym (Year)	Language
Culture Change Toolbox [68].	BC Patient Safety & Quality Council—BCPSQC (2017)	English
Action Planning Tool for the AHRQ Surveys on Patient Safety Culture [69].	Agency for Healthcare Research and Quality—AHRQ (2016)	English
Safer Together: A National Action Plan to Advance Patient Safety [70].	Institute for Healthcare Improvement—IHI (2020)	English
Sentinel Event Alert 57: The essential role of leadership in developing a safety culture [71].	Joint Commission International—JCI (2021)	English
The Incident Decision Tree: Guidelines for Action Following Patient Safety Incidents [72].	National Patient Safety Agency—NPSA (2005)	English
Global Patient Safety Action Plan 2021–2030 [1].	World Health Organization—WHO (2021)	English

**Table 4:** Presentation of strategies mapped in the literature.

**Recommendation [Reference]**	**Concept**	**Frequency**
Organizational Fairness [1,20,24,25,29,33,39,60,70]	It seeks to ensure that everyone is treated fairly and respectfully, with policies that promote harmonious relationships and a positive work environment. This includes providing resources and incentives for developing team skills.	9
Transparency [1,17,25,28,32,40,69,72]	Seeking to maintain a standard of openness in all aspects of patient care, especially in disclosing security incidents, is a key factor for trust and continuous improvement.	8
Teamwork [1,17,18,20,23,24,25,27,28,29,30,31,33,35,36,37,39,40,41,42,45,47,48,49,51,53,55,59,60,63,67,70,71,72]	Strengthening safety culture involves promoting high-quality teamwork, with an emphasis on collaboration, communication, and effective conflict resolution, aiming for effective care and patient safety.	34
Communication [1,6,17,18,22,23,24,25,28,29,30,31,32,34,35,36,37,38,39,40,42,45,46,47,49,51,52,53,54,55,56,58,59,60,61,63,65,66,69,70]	Improving communication within the organization is essential for resolving conflicts and promoting a positive culture in the workplace, contributing to patient safety.	40
Active Leadership [1,21,22,23,24,25,28,29,30,31,33,35,37,39,40,41,42,44,45,48,49,51,55,58,59,64,65,66,67,69,70,71,72]	Leaders’ commitment to patient safety is essential. They must make the organization’s efforts visible, align incentives, and ensure the appropriate allocation of resources.	33
Psychological Safety [1,17,25,28,34,37,38,40,51,60,61,70]	Creating an environment where professionals feel free to express ideas, make suggestions, and openly discuss safety issues is essential for preventing errors.	12
Trust [1,19,25,31,72]	An effective security culture is based on mutual trust, shared understanding of security, and the effectiveness of preventative measures.	5
Working conditions [1,19,22,29,33,37,39,44,45,51,53,69,72]	Working conditions, including adequate workload and access to necessary supplies, are critical to patient safety and worker well-being.	13
Just Culture [1,6,17,18,19,21,22,25,28,29,31,34,38,39,40,46,51,55,58,59,69,70,71,72]	It seeks to learn from mistakes rather than looking for culprits, focusing on modifying the system to prevent the recurrence of incidents.	24
Management Support [19,21,22,24,25,28,29,30,31,33,34,39,51,58,70,71]	Senior management is expected to demonstrate a firm commitment to safety, including integrating patients and families into care strategies.	16
Reporting System for Safety Events [6,17,18,19,20,21,22,23,25,28,29,31,32,34,37,41,46,49,51,57,58,62,70]	Implementing effective incident reporting systems is essential to identify and prevent risks to patient safety.	23
Personnel Sizing [22,33,36,45,53,70]	Ensuring well-sized and trained teams is essential to avoid errors resulting from work overload and promote a safe environment.	6
Care Transitions [39,45,48,65,70]	Ensuring effective and safe transitions of care between different levels or locations of care is paramount to the continuity of care.	6
Patient and Family Engagement [19,25,29,32,36,44,48,50,51,53,65,71]	Encouraging the active participation of patients and families in the care process improves the safety and quality of care.	12
Education and Training [17,19,22,23,24,25,28,29,30,32,33,34,35,36,37,39,40,42,43,48,49,51,53,55,58,59,60,61,63,65,67,71,72]	Continuous training of healthcare professionals in patient safety practices is essential for promoting a safety culture.	32
Culture Assessment [1,19,26,29,30,32,33,35,36,47,48,50,51,55,59,62,63,65,67,72]	Regularly evaluating the security culture allows you to identify areas for improvement and implement effective strategies.	21
Cause Analysis [18,21,25,28,31,49,54,55,58]	Conducting root cause analyzes on security incidents contributes to understanding the underlying factors and implementing improvements.	9
Feedback on Reported Bugs [18,28,29,32,35,37,50,51,72]	Providing feedback on reported errors is important to encourage reporting and promote organizational learning.	9
Organizational Protocols [19,20,23,26,27,29,30,31,39,51,53,62,72]	Adherence to established patient safety protocols is essential to prevent errors and ensure safe care.	13
Investment in New Technologies/Equipment [22,25,29,38,39,43,50,51,67]	It is essential to provide financing for projects, including the implementation of new information technologies, to improve clinical practice and increase patient safety and satisfaction. Lack or malfunction of equipment are frequent causes of losses for both patients and professionals.	9
**Action [Reference]**	**Goal**	**Frequency**
CRM (Crew Resource Management) [18,30,49,58]	Utilize team resources and skills to manage critical situations effectively.	4
CIRS (Incident Reporting System) [18,58]	Report and analyze incidents, aiming for security improvements.	2
Simulation [1,18,29,47,55,59,60,63]	Conduct practical training that simulates real situations to improve skills and emergency response.	8
10-for-10 [18]	Pause for reflection and risk assessment before procedures.	1
Benchmarking [19]	Compare practices and performance to identify areas for improvement.	1
Walkrounds [1,20,37,52,53,59,66]	Conduct visits to the workplace, by leaders, to discuss safety and identify risks.	7
Meetings/Group dynamics [1,21,23,31,35,41,47,53,58]	Promote communication, cooperation, and conflict resolution within the team.	9
TeamSTEPPS [24,30,42,47]	Carry out a program focused on communication and teamwork for patient safety.	4
Swiss Cheese Model [25,28,36,39]	Take an approach to understand how errors happen and prevent them.	4
Agile Methodologies (Lean) [25,27,41,51,67]	Carry out methodologies focused on efficiency, eliminating waste, and improving processes.	5
Employee Safety Pulse [31]	Conduct regular surveys to assess safety perceptions among employees.	1
Team Engagement [31,33,35,41,50,51,55,57,58,60,67,72]	Encourage everyone’s active participation in promoting safety.	12
PDSA/Demming cycle [20,32,48,51,62,67]	Create structure for testing and implementing continuous improvements.	6
No Brief, No Start [34]	Hold meetings to avoid starting procedures without a prior briefing to align the team.	1
Learning with Excellence/Sharing Positive Experiences [34]	Focus on successes to motivate and educate the team.	1
Speaking Up [38,61]	Encourage the expression of safety concerns in the moment.	2
Zen Rooms [45]	Create rest spaces for the well-being of healthcare professionals.	1
Healthy for Good [45]	Create health and well-being programs for professionals.	1
Bedside shift change [45,48]	Involve patients and families in the transfer of information.	2
Bundle [51]	Disseminate a set of evidence-based practices to improve care outcomes.	1
CUSP [59]	Implement an approach to improve safety focused on the healthcare team.	1
Step Back/Process Review [60]	Evaluate and improve work processes.	1
Transformational Leadership [64]	Lead and motivate teams to achieve high safety standards.	1
NetworkZ [63]	Create a team simulation based on improving communication and patient safety.	1
Critical Language [68]	Use clear communication in high-risk situations to ensure understanding.	1
Feedback [24,68]	Encourage a culture of requesting and receiving feedback for continuous improvement.	2
Learning Boards [68]	Create visual tools to share lessons learned and best practices.	1
Learning from Defects [17,21,68]	Analyze and learn from mistakes to avoid repetition.	3
Reporting Culture [17,21,22,68]	Encourage incident reporting to learn and improve.	4
Security Tutor [68]	Assign team members to focus on aspects of security.	1
Telephone [68]	Use direct communication to clarify doubts and convey urgent information.	1
Use First Names [68]	Promote a more personal and less hierarchical environment.	1
TRIZ [68]	Solve problems creatively.	1
25 Gets You 10 [68]	Create strategies to prioritize ideas or problems to be solved.	1
5 Whys [68]	Finding the root cause of a problem	1
Collaboration [24]	Establish joint work and knowledge sharing.	1

## Data Availability

The datasets used during and/or analyzed during the current study are available from the corresponding author upon reasonable request.
